# Influence of Zr Microalloying on the Microstructure and Room-/High-Temperature Mechanical Properties of an Al–Cu–Mn–Fe Alloy

**DOI:** 10.3390/ma17092022

**Published:** 2024-04-26

**Authors:** Jingbin Liu, Jingyi Hu, Mengyu Li, Guiliang Liu, Yuying Wu, Tong Gao, Shushuai Liu, Xiangfa Liu

**Affiliations:** Key Laboratory for Liquid-Solid Structural Evolution and Processing of Materials, Ministry of Education, Shandong University, 17923 Jingshi Road, Jinan 250061, China; 18553951918@163.com (J.L.); 202320616@mail.sdu.edu.cn (J.H.); 15634101153@163.com (M.L.); liuguiliang109@sdu.edu.cn (G.L.); wuyuying@sdu.edu.cn (Y.W.); xfliu@sdu.edu.cn (X.L.)

**Keywords:** Al–Cu–Mn–Fe alloy, microstructure, microalloying, high-temperature strength

## Abstract

Here, 0.3 wt.%Zr was introduced in an Al-4 wt.%Cu-0.5 wt.%Mn-0.1 wt.%Fe alloy to investigate its influence on the microstructure and mechanical properties of the alloy. The microstructures of both as-cast and T6-treated Al–Cu–Mn–Fe (ACMF) and Al–Cu–Mn–Fe–Zr (ACMFZ) alloys were analyzed. The intermetallic compounds formed through the casting procedure include Al_2_Cu and Al_7_Cu_2_Fe, and the Al_2_Cu phase dissolves into the matrix and re-precipitates as θ′ phase during the T6 process. The introduction of Zr results in the precipitation of L1_2_-Al_3_Zr nanometric precipitates after T6, while the θ′ precipitates in ACMFZ alloy are much finer than those in ACMF alloy. The L1_2_-Al_3_Zr precipitates were found coherently located with θ′, which was assumed beneficial for stabilizing the θ′ precipitates during the high-temperature tensile process. The tensile properties of ACMF and ACMFZ alloys at room temperature and elevated temperatures (200, 300, and 400 °C) were tested. Especially, the yield strength of ACMFZ alloys can reach 128 MPa and 65 MPa at 300 °C and 400 °C, respectively, which are 31% and 33% higher than those of ACMF alloys. The strengthening mechanisms of grain size, L1_2_-Al_3_Zr, and θ′ precipitates on the tensile properties were discussed. This work may be referred to for designing Al–Cu alloys for application in high-temperature fields.

## 1. Introduction

In the past decades, with the development need for lightweight materials, aluminum alloys have been widely used in automotive, aerospace, military, and other fields due to their excellent properties, such as low density and high specific strength [1,2,3]. However, high-strength aluminum alloys are still confined to low-temperature environments below 150 °C in industrial applications, falling short of modern industrial requirements for the development of heat-resistant and high-strength aluminum alloys at 300 °C to 400 °C [4,5,6]. At present, the typical common heat-resistant casting aluminum alloys include Al–Si–Cu–Ni–Mg and Al–Cu alloys. Al–Si–Cu–Ni–Mg alloys are mainly used in the automotive parts manufacturing field, such as pistons [7]. Relatively speaking, Al–Cu cast alloys have low density, good heat resistance, and potential high-temperature stability [8,9,10], making them one of the most promising aluminum alloy series for high-temperature applications in wider areas [11,12,13,14]. For example, the reported yield strength (YS) of Al–Cu 206 alloy after T7 treatment can be up to 390 MPa at room temperature (RT) and remains at 93 MPa at 300 °C [15]. The precipitation sequence in Al–Cu alloys is supersaturated solid solution → G.P.I zone → G.P.II zone (θ″) → θ′ → θ [16], where the sub-stable θ′-Al_2_Cu, as the main precipitation phase, produces precipitation hardening, which gives the alloys excellent strength [17,18]. However, when the temperature exceeds 250 °C, the θ′-Al_2_Cu precipitated phase coarsens dramatically and transforms into the stable equilibrium phase θ-Al_2_Cu, which loses the strengthening effect [19]. How to suppress the coarsening of the θ′ precipitated phase at high temperatures becomes one of the key issues in strengthening the high-temperature properties of aluminum alloys.

Improving the casting properties of alloys plays an important role in increasing the heat resistance of alloys. One method is to set up barriers at grain boundaries by introducing a second phase with a high melting point, which hinders the sliding of the grain boundaries. This method can be achieved by adding rare earth elements (Ce, Er, Yb, Gd, La, and Y) or alloying with transition elements (Fe, V, Cr, and Mo) to form Al-TM alloys, such as Al–Cu–Ce [20], Al–Cu–Er [21], Al–Cu–La [22], Al–Cu–Y [23], Al–Cu–Yb [24], Al–Cu–Gd [24], and Al–Fe–V–Si [25]. Amer et al. [26] reported that Al–Cu–Er–Mg–Zr–Cr–Fe–Si–Ti alloys with 0.2 wt.%Cr addition were able to reach YS of 250 MPa and 225 MPa at 200 °C and 250 °C, respectively. These elements can react with Al, Cu, and Mg during the eutectic reaction to form complex intermetallic compounds, which have high melting points. These compounds can form closed or semi-closed rings at grain boundaries and have outstanding thermal stability, which significantly improves the casting properties and heat resistance of Al–Cu alloys.

In recent years, microalloying has become a popular and effective strategy for strengthening the high-temperature properties of aluminum alloys [27,28]. On the one hand, microalloying elements can make solute atoms in Al–Cu alloys polarize at the θ′ interface, causing solute resistance and lowering the interfacial energy to regulate the nucleation and growth of the Al_2_Cu phase, thus improving the high-temperature stability of the θ′ precipitated phase [23,29]. On the other hand, the nucleation of θ′ precipitates is promoted by adding microalloying elements to the Al–Cu alloy to form the L1_2_-Al_3_X dispersed phase with excellent thermal stability [30,31]. At present, the L1_2_-Al_3_X dispersed phase formed by the addition of Sc, Er, and Y is often reported [32,33]. For example, Yang et al. [34] reported that the creation of the L1_2_-Al_3_Sc phase enables Al–Cu–Sc alloys to achieve YS as high as 120 MPa at 300 °C. However, the price of Sc is very expensive, resulting in its application in the industrial production of Al alloys receiving great limitations. Compared to Sc, Zr is also a good candidate for enhancing high-temperature strength, as the Zr element can form the L1_2_-Al_3_Zr structure and is capable of good resistance to roughening at high temperatures of up to 400 °C, with good thermal stability, thus effectively improving the strength and heat resistance of the alloy [35]. For instance, Mondol et al. [36] have shown that the YS of the Al-4.5 wt.%Cu-0.48 wt.%Zr alloy can reach 198 MPa at 250 °C. Kumar Makineni et al. [37] have measured the quaternary Al-2 at.%Cu-0.1 at.%Nb-0.15 at.%Zr with a YS of 250 MPa at 250 °C.

Furthermore, most of the researchers reported the presence of more impurity elements in binary Al–Cu alloys, such as Fe. The small solid solubility of Fe in Al–Cu alloys leads to the precipitation of hard and brittle Fe-rich intermetallic compounds, such as Al_6_(Fe, Mn) and Al_7_Cu_2_Fe(β-Fe) [38,39], which greatly reduces the mechanical properties of the alloys (e.g., ductility and fatigue properties) [40,41]. The content of Fe in high-performance Al–Cu alloys is usually limited to 0.15 wt.% [42], so we designed a Fe content of 0.1 wt.%. Mn is a commonly added element in Al–Cu casting alloys because Mn can transfer the platelet Fe-rich intermetallic compounds into the Chinese script, thus reducing the deleterious effects of Fe elements on mechanical properties [38,39,43]. Generally, 0.4 wt.%-1.0 wt.%Mn is added to Al–Cu alloys to minimize the harmful effects of Fe [41]. Meanwhile, Mn can form fine, diffusely distributed rod-like T_Mn_ (Al_20_Cu_2_Mn_3_) in Al–Cu alloys after solid solution treatment, which has excellent thermal stability properties and can be used as a heat-resistant alloy-reinforcing phase to improve the strength of the material at room temperature and high temperature [44,45]. Therefore, 0.5 wt.% of Mn was designed in this study. Besides, the operating temperature of the top of the piston in modern automobiles is above 350 °C, and the peak stress can reach more than 20 MPa [46]. While most of the current studies focus on below 300 °C, there are even fewer studies above 350 °C. It has been shown that the addition of Zr or Mn only improves the stability of the alloy at 200 °C and 300 °C, respectively [13]. 

In this paper, multi-component Al–Cu–Mn–Fe alloys, and the strengthening mechanism of Zr to improve the strong plasticity of Al–Cu-based alloys at room and high temperatures up to 400 °C, are investigated. The microstructure of as-cast and T6-treated alloys is studied, and the strengthening mechanisms of Zr are discussed.

## 2. Materials and Methods

### 2.1. Fabrication of Alloys

In this experiment, commercial pure Al (99.99 wt.%), Al–Cu, Al–Mn, Al–Fe, and Al–Zr master alloys were used to fabricate nominal multi-component alloys: Al-4 wt.%Cu-0.5 wt.%Mn-0.1 wt.%Fe, without or with 0.3 wt.%Zr, named as ACMF and ACMFZ, respectively. The composition of the two alloys was detected using an optical emission spectrometer (Spectra Max, PE8000; Molecular Devices, San Jose, CA, USA), and the results are shown in Table 1. Firstly, a total of 3 kg of pure aluminum and other master alloys was weighed and placed into clean clay-bonded graphite crucibles and melted by a medium-frequency induction furnace at 790 °C for 3.5 h. Then, 0.5 wt.%Al-5Ti-1B refiner was added and 0.5 wt.%C_2_Cl_6_ was used for slag removal and degassing of the melt. Finally, the melt at 790 °C was poured into a bar cast iron mold with sprue preheated at 300 °C for 4 h to obtain both alloys. The bars obtained from the cast iron molds had a diameter of 20 mm. After solidification, the as-cast ingots were subjected to T6 heat treatments. In this case, both as-cast ingots were solution-treated at 520 °C for 24 h, then removed and immediately quenched in water, and finally aging-treated at 185 °C for 0–74 h and cooled in air. Both alloys were heat-treated simultaneously in the same heat treatment furnace to ensure uniformity in the heat treatment conditions of the alloys.

### 2.2. Microstructure Analysis

Metallographic samples were prepared using wire-cutting and hot inlay mechanisms, and then sanded by different grit sizes of SiC sandpaper and polished with MgO solution. The microstructural analysis was carried out by field emission scanning electron microscopy (FESEM, SU-70; Hitachi, Tokyo, Japan), which was equipped with energy-dispersive X-ray spectrometry (EDS). The phase analysis of the alloys was implemented by using X-ray diffraction (XRD, Rigaku D/max-rB, Tokyo, Japan) with Cu Kα radiation: the scanning operating voltage and current were 40 kV and 100 mA, respectively, the scanning speed was 0.2°/min, and the scanning range was 10–90°. The statistical distribution of grain size was studied using an optical micrograph (OM, Leica DM2700M, Wetzlar, Germany) and a field emission scanning electron microscope (SEM, FEI Apreo 2C; FEI Company, Hillsboro, OR, USA) equipped with an EBSD detector (EDAX Velocity Super, Pleasanton, CA, USA) at 20 kV, and commercial software Channel 5 processed the results of the EBSD test. Analysis of microstructural details and precipitated phases after aging treatment was performed using a transmission electron microscope (TEM, FEI Talos F200X, with an accelerating voltage of 200 kV), which was equipped with EDS and selected area electron diffraction (SAED). The alloy samples were mechanically ground to a thickness of about 50 μm, and the samples were punched out with a punching machine to produce round samples with a diameter of 3 mm, and then thinned by a Precision Ion Polishing System (Gatan 695, Pleasanton, CA, USA) until the center perforation reached the requirement for observation.

### 2.3. Mechanical Testing

**Figure 1:**
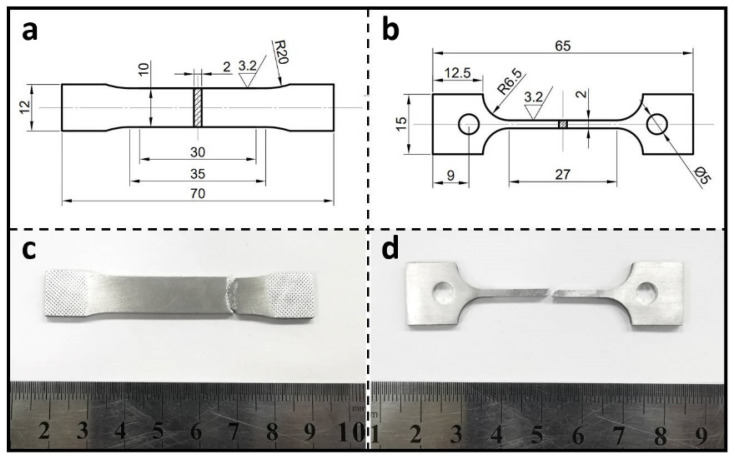
The dimensions of the tensile samples for the room-temperature (**a**,**c**) and high-temperature (**b**,**d**) tensile tests.

Brinell hardness measurements of the two alloys were tested using an HBS–3000 digital Brinell hardness tester (Hua Yu Zhong Xin, Laizhou, China)with an indenter diameter of 5 mm and a loading force of 2452 N, with each reported value being the average of five measurements. Room- and high-temperature plate tensile tests were carried out on both alloys in an electrically operated high- and low-temperature tensile testing machine (zwick-z250; Zwick Roell Group, Ulm, Germany) equipped with a laser extensometer (capable of measuring deformations in the range of scale distances from 1.5 to 120 mm), and all tensile tests were performed at a rate of 1 mm/min. For high-temperature tensile tests at 200 °C, 300 °C, and 400 °C, the specimens were placed in a resistance furnace, heated to the specified temperature, and then kept warm for 10 min before stretching. At each temperature, three specimens of each alloy were tested. The dimensions of the tensile samples for the room- and high-temperature tensile tests are shown in Figure 1.

## 3. Results

### 3.1. Microstructure Characterization for the ACMF and ACMFZ

**Figure 2:**
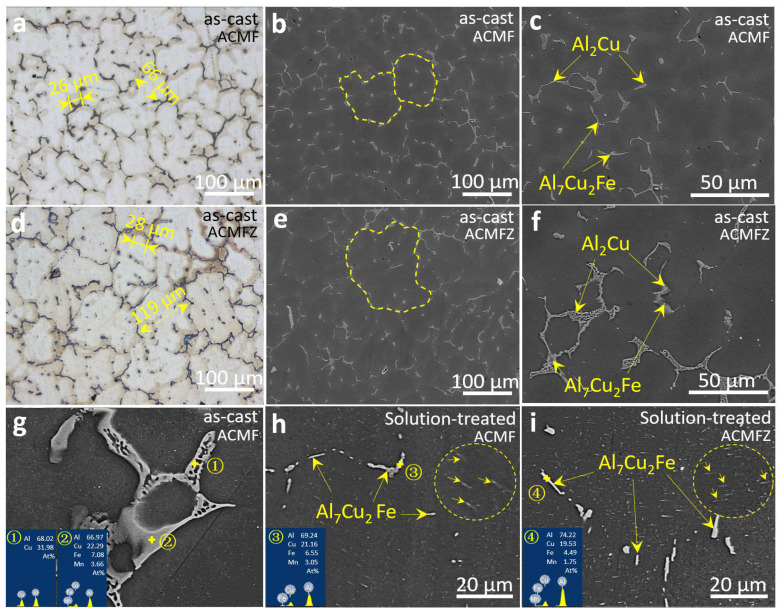
OM and SEM images of the as-cast and solution-treated ACMF (**a**–**c**,**g**,**h**) and ACMFZ (**d**–**f**,**i**) alloys: (**a**,**d**) OM images of as-cast ACMF and ACMFZ alloys. (**b**,**c**,**g**,**h**) SEM images and EDS analysis of as-cast and solution-treated ACMF alloy, in which the dotted lines in (**b**) represent twoα-Al grains, and the arrows in the circle of (**h**) represent precipitates. (**e**,**f**,**i**) SEM images and EDS analysis of as-cast and solution-treated ACMFZ alloy, in which the dotted line represents an α-Al grain, and the arrows in the circle of (**i**) represent precipitates.

**Figure 3:**
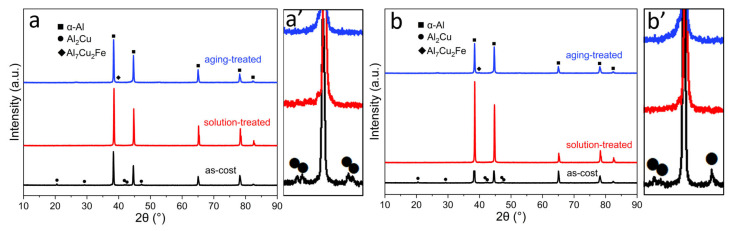
XRD patterns for the ACMF (**a**,**a’**) and ACMFZ (**b**,**b’**) alloys, as-cast, solution-treated, and aging-treated.

Figure 2 shows the typical microstructures and EDS analysis of ACMF and ACMFZ alloys. As marked in Figure 2a,d, the typical dendrite arm spacings were ~26 μm for the as-cast ACMF alloy and ~28 μm for the as-cast ACMFZ alloy, while the typical grain sizes were ~66 μm and 119 μm, respectively. This means that the inoculation of Zr led to matrix grain coarsening, which was confirmed by EBSD and discussed in the following text. Figure 2b,e show the SEM images of the two alloys, and the magnified images are presented in Figure 2c,f. From Figure 2c,f, it can be observed that the two alloys consisted of matrix phases in the gray region and intermetallic compounds in the bright region. Based on the EDS analysis shown in the inset of Figure 2g, it can be determined that the bright regions were eutectic Al_2_Cu and Al_7_Cu_2_Fe intermetallic phases [38,39], which was confirmed by XRD in Figure 3a,b, and the boundaries of the two phases were fused. Comparing the observations in Figure 2c,h, it can be found that the eutectic Al_2_Cu phase in the ACMF alloys disappeared after the solid solution treatment, and only the Al_7_Cu_2_Fe phase remained. The same results also appeared in Figure 2f,i, which indicates that the Al_2_Cu phases in both alloys in the as-cast state were basically dissolved in the α-Al matrix after the solid solution treatment. Meanwhile, a number of needle-like precipitates precipitated in the matrix could be observed after heat treatment in the enlarged area of Figure 2h,i, which will be characterized in detail via the TEM technique in the following.

In order to further prove that the eutectic Al_2_Cu phase disappeared after solid solution, XRD analysis was carried out. Figure 3 shows XRD patterns of the ACMF and ACMFZ alloys as-cast, solution-treated, and aging-treated, respectively. As can be seen, the diffraction peak of the Al_2_Cu phase was dissolved, and its diffraction peak disappeared after solid solution treatment in Figure 3a,b, indicating that Al_2_Cu was essentially a solid solution in the Al matrix. In addition, Figure 3a’,b’ display the magnified patterns in the squared area. It was found that the changes in the Al_2_Cu phase peaks could be seen more clearly, and it was also observed that the peaks of the α-Al phase were displaced to the right after the solid solution treatment compared to the cast state, indicating that the solid solution treatment promoted the dissolving of the Cu element in the α-Al matrix [47].

**Figure 4:**
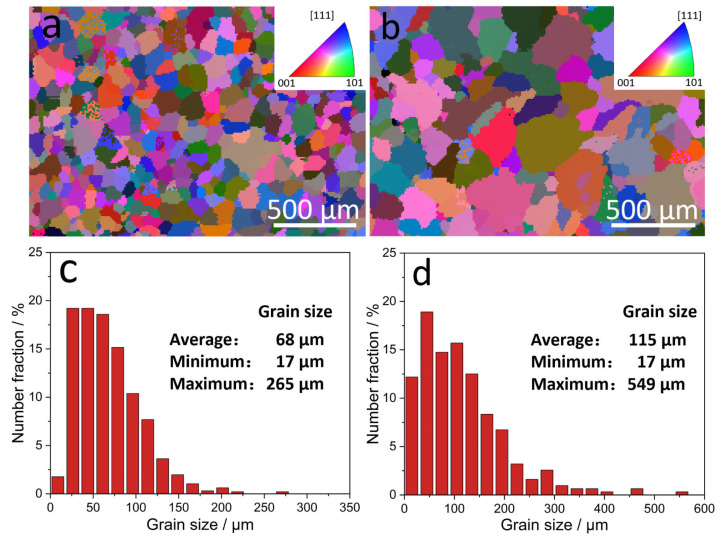
Electron backscatter diffraction (EBSD) images and statistical grain size distribution for the ACMF (**a**,**c**) and ACMFZ (**b**,**d**) alloys after T6 treatment.

The grain size of the α-Al matrix was described in order to better show the effect of Zr element addition on the grain size. Figure 4 shows the grain size of ACMF and ACMFZ alloys quantified by using the results of EBSD. Interestingly, it can be seen from Figure 4a,b that the grain size of the ACMFZ alloy was coarser compared to that of ACMF alloy grains, as mentioned above in Figure 2a,d. As is known, heat treatment did not significantly change the grain size of as-cast ACMF and ACMFZ alloys; therefore, this may be due to the addition of Al–Ti–B refiner, which resulted in finer grains in ACMF alloys, but ‘Zr poisoning’ occurred in ACMFZ alloys [48]. According to Fan et al. [49], TiB_2_ particles are not effectively nucleating substrates themselves, and they need the help of Al_3_Ti 2DC (two-dimensional compound) on their surfaces to nucleate α-Al during the solidification process. The mechanism of ‘Zr poisoning’ is due to the dissolution of Al_3_Ti 2DC on the surface of TiB_2_ in the Al–5Ti–B refiner and the generation of Ti_2_Zr 2DC. The Ti_2_Zr 2DC poorly matches with the crystallography of α-Al and thus weakens the nucleating ability of TiB_2_ particles [48]. For further characterization, Figure 4c,d quantitative statistics for the grain size distribution of the two alloys in the as-cast state showed that the average grain size of the ACMF alloy was about 68 μm, while that of the ACMFZ alloy was about 115 μm. Meanwhile, it was found that most of the grains in the ACMF alloy were smaller than 125 μm, and the largest grain size was 265 μm, while most of the grains in ACMFZ alloys were concentrated at a size of less than 200 μm, and the largest grain size reached 549 μm. This indicates that the occurrence of the ‘Zr poisoning’ phenomenon made the size of α-Al matrix grains larger. Therefore, scholars should pay attention to when Zr is used for microalloying when Al–Ti–B grain refiner is used. There is no doubt that the development of new grain refiners for application in Zr-containing Al alloys is urgent since a lot of Al alloys (e.g., 5182 and 7055 alloys) contain Zr, which will not be discussed in detail in this work.

**Figure 5:**
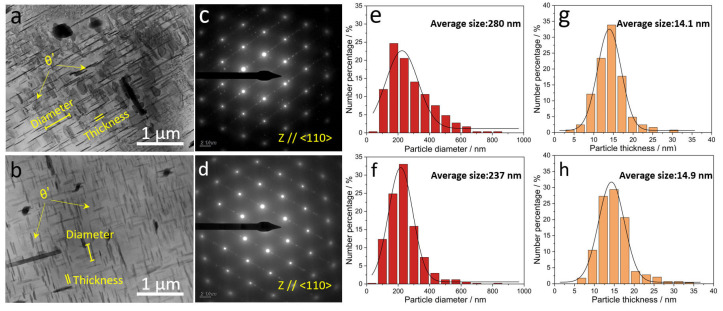
TEM images, selected area electron diffraction (SAED) patterns, and statistical size distribution for θ′(Al_2_Cu) precipitates in the ACMF (**a**,**c**,**e**,**g**) and ACMFZ (**b**,**d**,**f**,**h**) after T6 treatment: (**a**,**c**) TEM image and SAED pattern of ACMF alloy. (**b**,**d**) TEM image and SAED pattern of ACMFZ alloy. (**e**,**f**) The diameter size distribution for θ′(Al_2_Cu) precipitates in the ACMF and ACMFZ, respectively. (**g**,**h**) The thickness size distribution for θ′(Al_2_Cu) precipitates in the ACMF and ACMFZ, respectively.

Figure 5 shows the TEM observations of the precipitates in ACMF and ACMFZ alloys. From Figure 5a,c and Figure 5b,d and their corresponding selected area electron diffraction results, it can be seen that a large number of needle-like θ′(Al_2_Cu) precipitates were uniformly distributed in the two alloys, which are the main precipitation-strengthened phases. Furthermore, in order to compare the differences in the precipitated phases between ACMF and ACMFZ alloys, the particle size distributions of the precipitated phases in the two alloys were quantitatively counted under the same heat treatment conditions, and the corresponding results are displayed in Figure 5e,f and Figure 5g,h, respectively. It was found that the average thickness values of the θ′ phase in ACMF and ACMFZ alloys were 14.1 nm and 14.9 nm, respectively, and the average particle size of the precipitates in ACMFZ was 237 nm, which is 15% smaller than that of the θ′ phase in ACMF alloys. In addition, a comparison of the full width at half maximum (FWHM) of the two curves in Figure 5e,f showed that the FWHM of the ACMFZ alloy was smaller than that of the ACMF, indicating that the size of the precipitates was concentrated around the average size and was more uniform. This suggests that the presence of the Zr element promoted the precipitation of θ′ phase.

**Figure 6:**
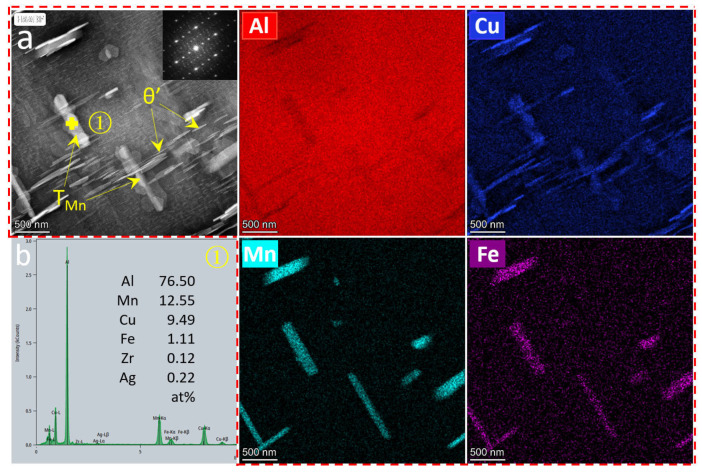
TEM images and EDS analysis for the ACMF (**a**,**b**) alloy after T6 treatment: (**a**) TEM images and Fast Fourier Transform image (FFT), and (**b**) EDS analysis of the rod-like precipitation particle.

**Figure 7:**
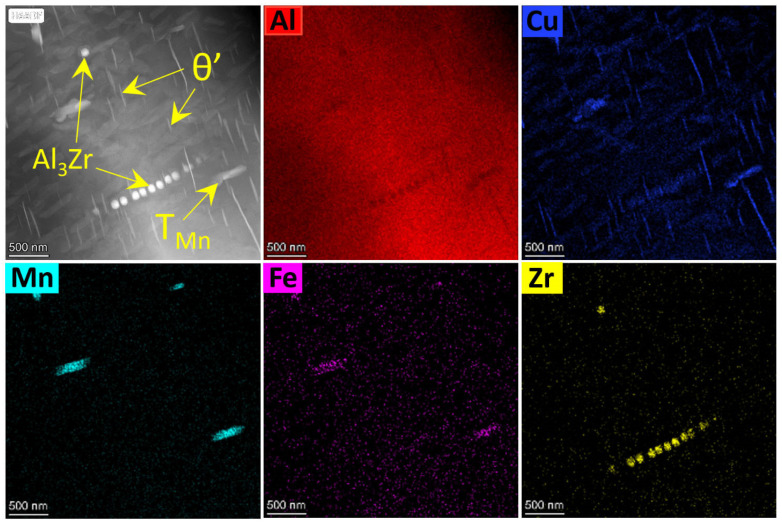
TEM images and EDS mapping analysis images for the ACMFZ alloy after T6 treatment.

Figure 6 and Figure 7 reveal the precipitated particle morphology of the ACMF and ACMFZ alloys. From Figure 6a, the EDS mapping result of the needle-like precipitated particles contained Al and Cu elements, which further confirmed they are θ′(Al_2_Cu). Meanwhile, it was seen that the rod-like precipitation particles also contained Al, Cu, Mn, and Fe elements, and the TEM-EDS analysis in Figure 6b shows that the Cu/Mn atomic ratio was close to 2/3. Chen et al. [50] and Liao et al. [44] found that there are rod-like precipitation particles in the solid solution state of Al–Cu–Mn and Al–Si–Cu–Mn alloys in the form of long or short rod-like precipitation particles, and they proved that these rods are in the form of T_Mn_ phase (Al_20_Cu_2_Mn_3_), with a Cu/Mn atomic ratio close to 2/3. Therefore, the identification of the rod-like particles in Figure 6a as the T_Mn_ phase was further confirmed by their Fast Fourier Transform (FFT) plots. This corresponds to the white needle-like precipitates in Figure 2h,i and the black rod-like precipitated phases in Figure 5a,b. As can be seen in Figure 7, the needle-shaped precipitated particles and the short-rod-shaped precipitated particles were identified as θ′(Al_2_Cu) and T_Mn_ phases, respectively, based on the EDS spectral results. The T_Mn_ phases of the two alloys were not significantly different. In addition, the EDS spectral analysis also showed the presence of another Zr-containing precipitated particle, which was spherical in shape. It was presumed to be L1_2_-Al_3_Zr composed of Al and Zr elements.

**Figure 8:**
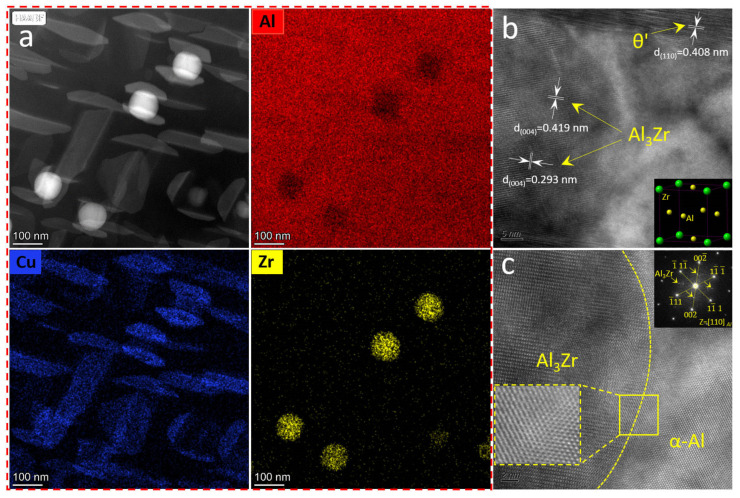
TEM images and high-resolution images for the ACMFZ alloy after T6 treatment: (**a**) TEM image and EDS mapping analysis images. (**b**) High-resolution analysis image and the unit cell structure of Al_3_Zr phase. (**c**) Interface between Al_3_Zr and α-Al, and FFT in the yellow area.

To further illustrate that this phase in the ACMFZ is the Al_3_Zr phase, the corresponding region was analyzed by EDS mapping and high-resolution analyses. Figure 8 shows the EDS mapping map, Fast Fourier Transform image (FFT), and high-resolution map in the ACMFZ. From the EDS mapping, it was seen that Figure 8a contains two phases: an Al_2_Cu phase composed of Al and Cu, and a spherical precipitate composed of Al and Zr elements. Figure 8b,c shows that the spherical precipitate is the Al_3_Zr phase, which was also characterized by the FFT of the yellow area in Figure 8c, and it exhibited a coherent relationship with the aluminum matrix in the magnified patterns in the squared area [13]. In addition, the Al_3_Zr phase has a face-centered cubic structure in Figure 8b, which is the same as the Al matrix. At the same time, it was observed that the Al_3_Zr particles were in contact with θ′(Al_2_Cu) in most cases, suggesting that the Al_3_Zr particles play an important role in the nucleation and growth of θ′(Al_2_Cu), leading to a refined distribution.

### 3.2. Mechanical Properties of the ACMF and ACMFZ

**Figure 9:**
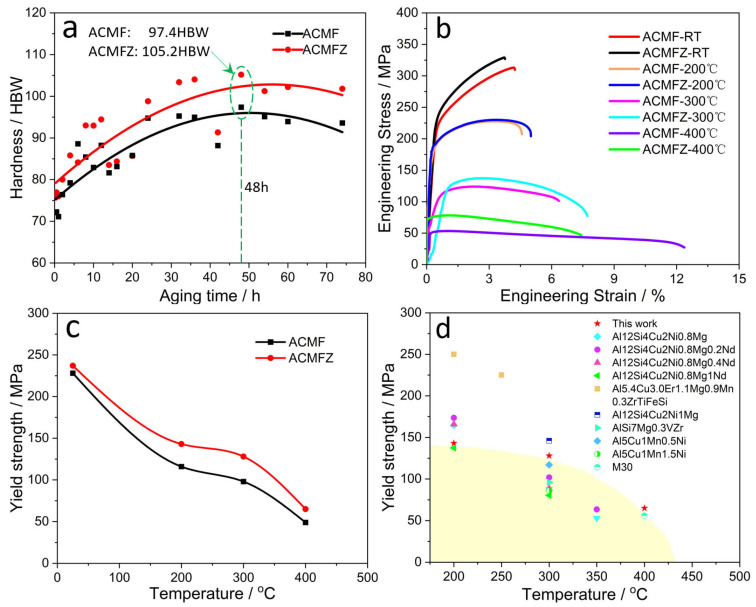
Mechanical properties for the ACMF and ACMFZ alloys after T6 treatment: (**a**) Brinell hardness of matrix, (**b**) tensile engineering stress–strain curves tested at different temperatures, (**c**) yield strength versus temperature, and (**d**) comparison between the yield strength of the ACMFZ alloy with heat treatment and that of reported alloys from 200 to 400 °C [26,51,52,53,54].

The hardness and mechanical properties of ACMF and ACMFZ alloys after T6 treatment were tested in Figure 9, respectively. Figure 9a shows the relationship between Brinell hardness and aging treatment time for ACMF and ACMFZ alloys, respectively. It was found that both alloys reached their maximum values at the aging treatment time (~48 h) when the Brinell hardness of ACMFZ was 105.2 HBW, which is 7.8 HBW higher than that of ACMF. This was mainly attributed to the presence of the L1_2_-Al_3_Zr phase and the increase in number density of the θ′ phase. Meanwhile, the Brinell hardness of both alloys increased with the aging time before the aging peak time, and their hardness decreased with the aging time after the aging peak time.

Figure 9b shows typical engineering stress–strain curves for the ACMF and ACMFZ alloys tested at RT, 200 °C, 300 °C, and 400 °C, and the corresponding numerical results are shown in Table 2. It was found that the ultimate tensile strength (UTS) and YS were improved from 311 MPa and 228 MPa to 329 MPa and 237 MPa at RT in Table 2, respectively. With the increase in temperature, the UTS and YS of the two alloys decreased slightly, accompanied by a significant increase in elongation (EL), indicating that high-temperature softening of the Al matrix occurred in both alloys. In addition, compared to the ACMF alloy, the UTS of the ACMFZ alloy was 228 MPa, 133 MPa, and 71 MPa at 200 °C, 300 °C, and 400 °C, which are 0%, 6%, and 31% higher than that of the ACMF alloy, respectively. Meanwhile, the YS of ACMFZ was 143 MPa, 128 MPa, and 65 MPa at 200 °C, 300 °C, and 400 °C, which are 23%, 31%, and 33% higher than the ACMF alloy, respectively. Figure 9c presents the decline of YS with variations in temperature. The rate of decrease of the ACMFZ alloy became gradually slower until 300 °C, which may be the reason for the excellent thermal stability of Al_3_Zr particles. Figure 9d exhibits a comparison of the YS between different alloys at elevated temperatures [26,51,52,53,54]. It was found that compared to other cast heat-resistant alloys, the ACMFZ alloy showed advantages at 300 °C and 400 °C. The result shows that small amounts of L1_2_-Al_3_Zr particles and fine θ′ precipitation led to an increase in the stress required for YS [55]. 

### 3.3. Discussion

ACMF and ACMFZ alloys are α-Al-rich alloys, with the θ′ phase as the reinforcing phase, while the L1_2_-Al_3_Zr phase is also present in ACMFZ alloys. Grain refinement can effectively enhance the strength of the alloys at RT, and the grain size strengthening can be expressed by the Hall–Petch formula, as follows [56]:

(1) $$\sigma_{s}=\sigma_{0}+Kd^{-\frac{1}{2}}$$

where $\sigma_{0}$ is the YS of the aluminum alloy, K is a constant associated with the material, and d is the average grain size of the aluminum alloy.

From the results of EBSD analysis in Figure 4, it can be seen that the average grain size of the ACMFZ alloy was 115 μm, while that of the ACMF alloy was 68 μm. The ACMFZ alloy grains became coarser due to the interaction of Zr with Al–Ti–B. According to the Hall–Petch formula, the coarser grains should have lower strength, but the addition of the Zr element made the reinforcing phase θ′ number density larger as well as smaller in size, which can be seen in Figure 5, and the simultaneous presence of nano-Al_3_Zr phases resulted in a slight enhancement in the strength of the ACMFZ alloy.

It can be seen in Table 2 that the YS of the ACMFZ alloy was 31% and 33% higher than that of ACMF alloy at high temperatures of 300 °C and 400 °C, respectively. This is speculated for a variety of reasons. On the one hand, due to the generation of viscous flow at grain boundaries at high temperatures, the occurrence of relative sliding of grains along the grain boundaries, and diffusional creep, the moderate coarsening of the grains rather improved the high-temperature strength of the material [57]. On the other hand, the main strengthening phase in Al–Cu alloys is θ′, and it has a plate-like shape. Since the traditional Orowan formula applies to spherical precipitates, researchers have proposed a modified Orowan formula for the plate-like θ′ phase [58]. Based on this, NIE and Muddle [59] developed a refined model for θ′ precipitates, which has been validated several times [60,61]. The equation is as follows:

(2) $$\sigma_{\theta^{\prime }}=\frac{MGb}{2\pi \sqrt{1-\upsilon }}\times \left\{1/\left[{\left(\frac{3.247}{fAN_{V}^{2}}\right)}^{\frac{1}{6}}-{\left(\frac{0.077fA}{N_{V}}\right)}^{\frac{1}{3}}-{\left(\frac{1.521f}{N_{V}A^{2}}\right)}^{\frac{1}{3}}\right]\right\}\times \ln\left\{\frac{1.063}{b}{\left(\frac{f}{N_{V}\sqrt{A}}\right)}^{\frac{1}{3}}\right\}$$

where M is the Taylor coefficient, G is the shear modulus of the aluminum phase, b is the Burgers vector of dislocations in the aluminum matrix, υ is the Poisson’s ratio of aluminum, A is the aspect ratio (diameter/thickness) of θ′ phase, f is the volume fraction of θ′ phase, and $N_{V}$ is the quantitative density of θ′ phase. The model shows that the number density increases with decreasing diameter, leading to an increase in strength, under the condition that the volume fraction of the plate θ′ phase remains constant [59,62]. From the TEM image in Figure 5, it was found that the thickness of the two alloys was approximately 14 nm, but the average diameter of the θ′ phase in the ACMFZ alloy was 237 nm, which is smaller than that of the ACMF alloy (280 nm). It follows that the ACMFZ alloy has a greater number density based on the model. Therefore, the number density of the θ′ reinforcing phases in the ACMFZ alloy was larger than that of the ACMF alloy, leading to a higher YS.

Further, the incorporation of Zr can form nano-Al_3_Zr particles, which were observed as a large number of finely dispersed L1_2_-Al_3_Zr dispersed phases in Figure 6. It has been shown that L1_2_-Al_3_Zr particles can be polarized to the coherent or semi-coherent interface formed between the Al matrix and θ′, which can be clearly seen in Figure 8. Normally, the interfacial excess can reduce the interfacial energy leading to the segregation [63,64,65,66], and the reduction of the interfacial energy provides a lower driving force for the coarsening of the θ′ precipitated phase, which achieves the inhibition of the coarsening and growth of the θ′ phase [67,68] and improves the stability of the precipitates. Thus, the solute polarization toward the θ′ interface greatly improves the stability of the microstructure and has a thermodynamic stabilizing effect [13].

Moreover, the Al_3_Zr dispersed phase formed during homogenization can also provide heterogeneous nucleation sites for the main strengthening phase [69]. For example, Makineni et al. [37] used phase-field simulations to demonstrate that the Al_3_Zr phase provides heterogeneous nucleation sites for the θ″ phase. Zhao et al. [70] also demonstrated that Al_3_Zr assists in η′ phase formation during aging. Therefore, we speculated that L1_2_-Al_3_Zr also acts as a heterogeneous nucleation site in ACMFZ alloys, providing nucleation sites for the θ′ phase, increasing the number density of the θ′ phase, and contributing to the thermal stability of the θ′ phase.

**Figure 10:**
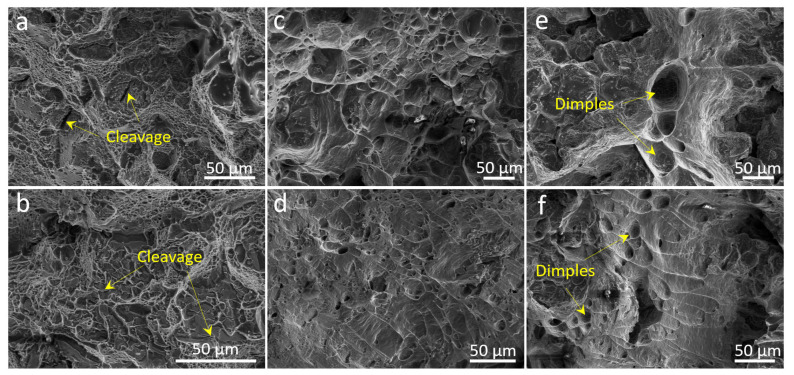
SEM images for tensile fracture surfaces of the ACMF (**a**,**c**,**e**) and ACMFZ (**b**,**d**,**f**) alloys: (**a**,**b**) RT, (**c**,**d**) 300 °C, and (**e**,**f**) 400 °C.

To further illustrate the high-temperature tensile mechanism of the ACMFZ alloys, Figure 10 shows the tensile fracture micrographs of the two alloys at RT, 300 °C, and 400 °C. It was found that the alloys exhibited a mixture of ductile and brittle characteristics, containing disintegrated surfaces, tough nests, and tear ridges. At the same time, there were also fish-scale-like features at high temperatures, which implies that significant plastic deformation occurred [71]. In Figure 10a,b, it is shown that many small and shallow toughness dimples were observed in both alloys at room temperature, along with the presence of many cracks, which suggests that the alloys underwent toughness behavior at RT [72,73]. Comparison of Figure 10c,d shows that the tough nests in the alloys became deeper and larger with the increasing temperature, tear ridges were clearly observed on the fracture surface, and the same phenomenon occurred at 400 °C, as in Figure 10e,f, which suggests that the alloys had a better elongation at high temperatures [62]. Due to the softening of the Al matrix and coarsening of the strengthening phases, the ACMF alloys had larger and deeper tough dimples and exhibited higher plasticity and lower strength than the ACMFZ alloys [62], which coincides with the tensile curves in Figure 9.

With the increasing demand for lightweight materials in the modern industrial sector, the need to develop heat-resistant and high-strength aluminum alloys has become even stronger, especially for high-strength aluminum alloys capable of operating in the 300 °C to 400 °C temperature range. In this study, the ACMFZ alloy containing L1_2_-Al_3_Zr particles showed excellent properties. For example, at 300 °C, the YS still reached 128 MPa and the elongation reached 7.7%. This is useful for the study of high-strength aluminum alloys in the temperature range of 300–400 °C and promotes the application in aerospace, automotive, and energy sectors.

## 4. Conclusions

(1) The ACMF alloy contained Al_7_Cu_2_Fe and Al_2_Cu phases. After solid solution treatment, the eutectic Al_2_Cu phase was incorporated into the α-Al matrix, and the fine θ′-Al_2_Cu phase appeared after aging treatment, which played a major role in strengthening. The L1_2_-Al_3_Zr phase was also generated in the ACMFZ alloy due to the addition of Zr. Meanwhile, the grain size of the ACMF alloy was 68 μm, and due to the interaction between Zr and the Al–5Ti–1B refiner, the grain size of the ACMFZ alloy was 115 μm, which became coarser.
(2) Hardness testing showed that the optimum aging process time for the two alloys was 48 h. Tensile tests at RT showed that the YS of the ACMF and ACMFZ alloys were 228 MPa and 237 MPa, respectively, with a slight increase for the ACMFZ alloy. This discrepancy was mainly due to the coarsening of the ACMFZ alloy grain caused by the introduction of the Zr element. However, the addition of Zr simultaneously generated the nano-L1_2_-Al_3_Zr particles in the ACMFZ alloy, which ultimately led to a slight enhancement in the ACMFZ alloy relative to the ACMF alloy.
(3) The YS of the ACMFZ alloy was 128 MPa and 65 MPa at 300 °C and 400 °C, respectively, which is enhanced by 31% and 33% compared with that of the ACMF alloy. The enhancement of the high-temperature strength was speculated to be due to the fact that the incorporation of Zr can form the L1_2_-Al_3_Zr precipitated phase. The L1_2_-Al_3_Zr precipitated phase can provide a heterogeneous nucleation location for θ′ phase after aging treatment, reducing the interfacial energy and enhancing the number density of the θ′ phase.
(4) There is an increasing demand in modern industry for the development of high-strength and heat-resistant aluminum alloys, especially at 300–400 °C. The present experiments illustrated that the addition of slow-diffusing Zr elements to Al–Cu–Mn alloys produces a well-stabilized, nano-precipitated phase. This further validated the feasibility of the microalloying strategy with high-solubility and slow-diffusing solutes in forming well-stabilized, nano-precipitated phases. This will contribute to the future application of high-strength and heat-resistant aluminum alloys in aerospace, automotive, and new energy fields. Meanwhile, the concept of introducing Zr into multicomponent Al–Cu–X alloys is feasible to enhance the high-temperature properties, while the interaction between Zr and the grain refiner or the X element should be taken into consideration.

## Figures and Tables

**Table 1:** Compositions of the as-cast ACMF and ACMFZ alloys (wt.%).

Alloy	Cu	Mn	Fe	Zr	Al
ACMF	3.92	0.583	0.106	-	Bal.
ACMFZ	4.14	0.532	0.103	>0.25	Bal.

**Table 2:** Mechanical properties of the ACMF and ACMFZ alloys.

Tensile Test Temperatures	Alloys	UTS (MPa)	YS (MPa)	EL (%)
RT	ACMF	311 ± 18	228 ± 2	4.2 ± 1.7
	ACMFZ	329 ± 1	237 ± 3	3.8 ± 0.4
200 °C	ACMF	229 ± 1	116 ± 21	4.6 ± 0.8
	ACMFZ	228 ± 2	143 ± 23	5.0 ± 1.0
300 °C	ACMF	125 ± 1	98 ± 16	6.4 ± 1.8
	ACMFZ	133 ± 4	128 ± 3	7.7 ± 3.9
400 °C	ACMF	54 ± 1	49 ± 1	12.4 ± 2.9
	ACMFZ	71 ± 7	65 ± 5	7.4 ± 1.1

## Data Availability

Data are contained within the article.
